# Evaluating the Clinical Reasoning Capabilities of AI Language Models in Diagnosing and Treating Depression

**DOI:** 10.1192/j.eurpsy.2025.1439

**Published:** 2025-08-26

**Authors:** V. W. L. Mok, S. N. Sasidharan, N. Q. Y. Chua, C. S. Lim

**Affiliations:** 1 Psychological Medicine, Changi General Hospital, Singapore, Singapore

## Abstract

**Introduction:**

Artificial intelligence (AI) language models are increasingly accessible tools that offer potential support in mental health care. Despite their promise in revolutionizing mental health care through symptom assessment and treatment suggestions, concerns about their validity, accuracy, ethical considerations, and risk management persist. This study evaluates the clinical reasoning capabilities of two leading AI language models in assessing a clinical case vignette of Major Depressive Disorder (MDD).

**Objectives:**

To evaluate the diagnostic accuracy, risk assessment proficiency, and quality of treatment recommendations provided by ChatGPT and Claude when applied to a standardised clinical vignette of a case of MDD.

**Methods:**

A clinical vignette describing a 50-year-old male patient exhibiting symptoms consistent with MDD was presented to both ChatGPT 4o and Claude 3.5 Sonnet. The patient had significant cardiac disease, leading to unemployment, social withdrawal, and passive suicidal ideation. Both AI models were asked five identical questions regarding: (1) diagnosis, (2) severity assessment, (3) first-line treatment recommendations, (4) optimal antidepressant selection, and (5) suicide risk evaluation. Two psychiatrists independently reviewed the responses for accuracy, comprehensiveness, and alignment with established guidelines and evidence-based treatment for depression with comorbid cardiac disease.

**Results:**

Both AI models correctly diagnosed MDD and accurately recognized the severity of the case due to the presence of suicidal ideation and significant functional impairment. Both offered comprehensive treatment recommendations, including pharmacotherapy and psychotherapy, and specifically suggested Sertraline as the antidepressant of choice due to its favourable cardiac safety profile. Both models assessed the patient as having a moderate to high suicide risk and provided a reasonably thorough analysis of risk and protective factors. However, limitations were noted in their ability to incorporate individualized patient nuances and psychosocial factors fully.

**Conclusions:**

ChatGPT 4o and Claude 3.5 Sonnet demonstrated significant capabilities in clinical reasoning, providing diagnoses and treatment recommendations that align with best clinical practices. Their responses were largely accurate and comprehensive, indicating potential utility as supportive tools for healthcare professionals. AI models may assist non-specialists in preliminary assessment and management but are not substitutes for professional psychiatric evaluation. Caution is advised in relying on AI for clinical decision making, and further refinement is necessary to enhance their ability to integrate patients-centered care and adherence to ethical guidelines, to mitigate risks associated with self-diagnosis and inappropriate treatment.

**Disclosure of Interest:**

None Declared

